# Effects of a prenatal mindfulness program on longitudinal changes in stress, anxiety, depression, and mother–infant bonding of women with a tendency to perinatal mood and anxiety disorder: a randomized controlled trial

**DOI:** 10.1186/s12884-023-05873-2

**Published:** 2023-07-31

**Authors:** Wan-Lin Pan, Li-Chiu Lin, Li-Yen Kuo, Mu-Jung Chiu, Pei-Ying Ling

**Affiliations:** 1 School of Nursing, National Taipei University of Nursing and Health Sciences, No.365, Ming-Te Road, Peitou District, Taipei, Taiwan; 2 Department of Nursing, HungKuang University, Taichung, Taiwan; 3 Department of Thanatology and Health Counseling, National Taipei University of Nursing and Health Sciences, Taipei, Taiwan; 4 Department of Nursing, Taiwan Adventist Hospital, Taipei, Taiwan; 5 Department of Obstetrics and Gynecology, Taiwan Adventist Hospital, Taipei, Taiwan

**Keywords:** Mindfulness, Perinatal care, Stress, Depression, Mother-infant bonding

## Abstract

**Background:**

Stress is a risk factor for poor physical and mental health, affecting new mothers’ ability, especially those with perinatal mood and anxiety disorders, to maintain their everyday lives. Over the past 50 years, global incidences of depression and anxiety disorders have increased, reaching pandemic levels. These incidences represent major public health issues that are challenging to detect and treat. Mindfulness programs are viable for reducing stress, anxiety, and depression. The present study evaluates mindfulness intervention effects on stress, anxiety, depression, and mother–infant bonding.

**Methods:**

We collected data on 102 women participating in a prenatal mindfulness program between July 2021 and March 2022; they were parallel and randomly assigned to experimental or control groups. The intervention group received an 8-week course in a prenatal mindfulness program, and the control group received usual standard prenatal care. The self-reported stress, pregnancy-related anxiety, and depression were assessed before and after the intervention and at 36 weeks of gestation. At 2 and 4 months postpartum, all participants provided self-reported their levels of stress, depression, and quality of mother-infant bonding.

**Results:**

Compared to the control group, the experimental group that received the prenatal mindfulness intervention experienced reduced prenatal stress, anxiety, and depression and reduced postnatal stress and depression. Despite this, there was no significant difference between the groups in terms of the quality of mother-infant bonding.

**Conclusions:**

Mindfulness prenatal programs are convenient and effective methods of decreasing stress, anxiety, and depression during the perinatal period. Based on our findings, prenatal mindfulness may play a role in mitigating mood and anxiety disorders and should be considered in future approaches to preventing psychological distress.

**Trial registration number:**

This trial has been prospectively registered at ClinicalTrials.gov (NCT04693130) and the first registration date was 12/24/2020.

## Background

The perinatal period is a time of high risk for developing mental illnesses, such as anxiety and depression disorders, which can occur during the antenatal period and up to one year after childbirth [1]. Perinatal mood and anxiety disorder (PMAD), commonly referred to as postpartum depression, is characterized by depression and anxiety disorders and is one of the most common disorders during pregnancy and the postpartum period [2]. Approximately 20% of first-time mothers experience varying degrees of PMAD. The estimated incidence of PMAD in Taiwan ranges from 17.2 to 43.1%, which seems higher than the global prevalence [3–5]. PMAD has been associated with neonate health problems, including preterm, intrauterine growth restriction, lower birth weight, and an increased risk of behavioral problems in the offspring [6–8]. In addition, mothers who experience higher perceived stress and emotional distress report inferior parenting abilities [9].

Mother-infant relationships refers to the primary emotional relationship between mother and infant. Particularly during the first year after childbirth, the quality of the relationship has been shown to directly affect the child’s physical, psychological, and developmental health [10]. Strong mother-infant bonding leads to positive outcomes throughout the life of the child, and mother-infant communication should be intimate, warm, stable, and kind, and provide comfort and joy for both the baby and the mother [11]. Untreated mood disorders can lead to feelings of guilt or incompetency as a mother or the inability to bond with the infant, and extreme cases can end in infanticide or suicide [12, 13]. However, because mental illness is often stigmatized or ignored, approximately half of people with PMAD are estimated to remain unidentified and, therefore, do not receive appropriate therapy [14].

Women with PMAD face many challenges when adapting to life changes, and the importance of facilitating adaptation should be emphasized when developing interventions. Sawyer, Kaim [15] pointed out that providing PMAD mothers with postpartum depression-related symptoms and parenting help within four weeks after delivery is particularly important. However, for Chinese women, this period is essential for engaging in postpartum confinement practices, in which the daily life of the postpartum mother is often supported by the woman’s mother or mother-in-law [16]. In recent years, due to social changes and the prevalence of small families, it has become common for women to stay in postpartum care centers or hire a yuesao (maternity caregiver specializing in caring for mothers and newborns during the postpartum confinement period [16]) after giving birth [17]. It is strongly recommended to pay more attention to the impact of cultural practices on mental health.

Mindfulness is defined as a state of deep awareness, which is consciousness characterized by clarity of focus, flexibility of attention, and a non-preconceived awareness of accepting events in the present moment [18, 19]. Mindfulness training is thought to promote adaptation through mind, body, and spiritual connections influenced by breathing and meditation [20]. A review article describes numerous studies that show negative relationships between mindfulness and psychological symptoms among women during pregnancy and the postpartum period [21]. Recent studies have indicated, however, that psychosocial education or behavioral intervention may not be effective in reducing mother–infant bonding disorders in women with PMAD conditions. Therefore, there is no opportunity to fully estimate effect sizes, making them unsuitable for valid statistical analysis [22–24]. There are limited empirical studies to support the claim that prenatal mindfulness-based interventions mitigate the quality of mother-infant bonding in the postpartum period.

As pregnancy is a major transition in a woman’s life, stress increases and mood disorders are common during pregnancy [25]. In Stone, Diop’s [26] study, 58% of women reported experiencing at least one stressor during pregnancy and the postpartum period, which contributed to the increased the prevalence of depression symptoms. Based on current evidence, non-pharmacological interventions that promote the well-being of peripartum women are of considerable importance. In recent years, mindfulness interventions have gained increased attention due to their effectiveness in reducing depression, anxiety, and stress [25].

Mindfulness refers to focusing one’s attention on the present moment, including physical sensations and sensory impressions (e.g., breathing, smelling, and hearing) experienced at that time. This practice helps individuals experience the moment rather than dwelling on negative emotions. Individuals can use cognitive reappraisal to reduce stress, decreasing psychological distress [27, 28]. For example, pregnant women are instructed to touch their own belly while breathing mindfully. It is through this approach that they are able to focus on their belly movement as well as the air entering and leaving their lungs. In contrast, postpartum women may be instructed to touch the crying baby while focusing on changes in the baby. Mindfulness skills development depends on awareness and intentional effort [29]. These exercises can be as simple as holding a baby quietly while maintaining eye contact in a gentle, loving manner. Deliberate mindfulness practice can help women more accurately interpret infants’ intentions and motivations, practice non-judgmental awareness of the baby’s behavior, and provide appropriate care for the baby to reduce the stress of looking after the baby [30, 31].

### Objectives

The aim of this study was to evaluate the effects of prenatal mindfulness intervention that can help women with PMAD overcome stress and psychological distress to promote a positive relationship with their infants in the postnatal period. The primary outcome is reduced perinatal mood distress. The secondary outcome is successful mother–infant bonding, which is also seen as the program’s long-term effect. Tertiary outcomes are obstetric data, especially on different types of postpartum confinement care, i.e., family care, yuesao care, and postpartum care centers.

## Methods

### Design and setting

This study adopted a two-arm, randomized longitudinal pre– and post-intervention design. Data were collected between July 2021 and March 2022. The study was approved by the Institutional Review Board of the study hospital and registered with ClinicalTrials.gov (NCT04693130).

### Participants

Women were recruited from an antenatal clinic at a teaching hospital in Taipei, Taiwan, using a convenience sampling method. Women who met the following criteria were included in the study: (1) age ≥ 20 years; (2) EPDS score ≥ 10; (3) singleton pregnancy at 13–28 weeks of gestation; and (4) willingness to participate in the mindfulness educational program. Women with high-risk maternal or fetal complications and psychiatric disorders were excluded.

Prior to recruitment, we asked potential participants using the Edinburgh Postnatal Depression Scale (EPDS) to indicate possible PMAD [1, 32, 33]. The EPDS was completed by 388 women, of whom 286 participants had low depressive symptoms (EPDS score < 10). Therefore, they were ineligible to participate in the study. In total, 102 women who met the inclusion criteria consented to random allocation, with some assigned to the intervention group (8-week prenatal mindfulness program) and others to the control group (standard care).

### Intervention

The intervention course was designed specifically for pregnant women with potential depression or anxiety symptoms. The intervention was adapted from Nancy Bardacke’s traditional mindfulness-based childbirth and parenting (MBCP) program, which was designed to prepare parents for childbirth and parenting [34]. For this study, the sessions were two hours long every week for the duration of the eight-week prenatal mindfulness curriculum. Discussions on the risk of PMAD were included in the session. We limited each group to 8–12 couples. Women and their partners were taught to use formal and informal mindfulness strategies to cope with stressful events during pregnancy, childbirth, and the postpartum period. The specific contents of this course include awareness of fetal/newborn, mindfulness practices for improved interpersonal relationships, techniques for coping with labor pains [34], and women with PMAD how to adapt to perinatal life [35] (Table 1). At each session, participants were asked to indicate how often and when they practiced formal and informal mindfulness meditations during the week. Participants were expected to consistently engage in 30-minute practice sessions six times per week.

**Table 1:** Overview of the 8-week perinatal mindfulness program intervention

Week	Goals	Practical exercise(s)	
1	Understand your inner workings during pregnancy Automated guidance		Eating a raisin with mindfulness meditation Breathing meditation with baby
2	Build a new life in awareness		Body scan Mindfulness principles Awareness of breathing and body
3	Talking about childbirth from the perspective of mind and body		Normal delivery process and childbirth Mindfulness of sounds and thoughts Mindfulness yoga during pregnancy
4	Facing aversion to pain		Ice cube exercise Mindful stretching and walking
5	Meditation to stay focused during childbirth		Mindfulness practices for coping with pain and touch practice Sitting meditation focusing on a difficult or stressful situation
6	Coping with difficulty Be the best teammate in childbirth		Mindfulness practices for coping with advanced pain Using the 3-min breathing space in stressful/painful situations
7	How can I best take care of myself and my baby?		My baby is the best mindfulness teacher Identifying postpartum depression triggers in the confinement periods
8	Using what has been learned to deal with future moods		Course review Keep a long-term meditation practice for postnatal life

The prenatal mindfulness program was delivered by two staff members who had previously taught MBCP together for five years. To reduce the potential drop-out rate during the research period, the experimental group was invited to join the mindfulness team communication tool and the freeware app LINE. Participation in LINE was only encouraged, but not required. The LINE provided weekly information regarding mindfulness pregnancy and parenting and allowed group members to interact with each other. The women in the intervention group and control group received standard antenatal care at the study hospital. This included regular antenatal examination, antenatal care counseling, delivery care, and postpartum nursing management.

### Measurements

Demographic variables, including age, education, marital status, and employment status et al. were collected. A repeated assessment design, including baseline before randomization (T0), post-intervention (T1), 36 weeks of gestation (T2), 2 months after childbirth (T3), and 4 months after childbirth (T4) was used to evaluate the effectiveness of the intervention on depression and stress symptoms. The testing time points were selected because they are typical PMAD screening time points [36]. Pregnancy anxiety effects were measured at T0, T1, and T2, while the quality of mother-infant bonding was assessed at T3 and T4. To reduce the risk of spreading COVID-19 during the outbreak, we reduced contact between people by collecting questionnaires T1 to T4 online, with only questionnaire T0 being collected face-to-face.

#### Edinburgh postnatal depression scale

The EPDS is designed to measure the intensity of depressive symptoms over the past week, using 10 items scored using a 4-point Likert scale. Each item is scored from 0 to 3, and the total score ranges from 0 to 40. Higher scores indicated more depressive symptoms, which resulted in good reliability (Cronbach’s α = 0.77) [37]. The EPDS was recommended as a PMAD symptom-screening tool [1]. Using a cutoff score of 10, 10–12 indicated “possible PMAD’’ and ≥ 13 indicated “PMAD” [32, 33].

#### Pregnancy-related thoughts questionnaire

Anxiety symptoms were assessed using the Pregnancy-Related Thoughts Questionnaire (PRT), which employs a 10-item scale to measure the frequency with which a person has worried or paid attention to baby health, labor, delivery, and caring for babies over the past 7 days. Each item uses a 4-point Likert scale, and the total score ranges from 10 to 40, with higher scores indicating more anxiety [38]. The PRT has acceptable reliability (Cronbach’s α = 0.81).

#### Perceived stress scale-10

Perceived stress was assessed using the Perceived Stress Scale-10 (PSS-10). The PSS-10 assesses perceived stressful experiences or stress responses that occurred during the past month, using a 5-point Likert scale (0 = never and 4 = very often). Total scores range from 0 to 40, with higher scores indicating greater perceived stress. Scores of 0–13 indicate ”low stress”, scores of 14–26 indicate “moderate stress”, and scores of 27–40 indicate “high stress” [39], with good reliability (Cronbach’s α = 0.83).

### Postpartum bonding questionnaire

The mother’s feelings or attitudes toward her baby were assessed using the Postpartum Bonding Questionnaire (PBQ), which is a 25-item scale designed to measure quality of mother-infant bonding. Each item is rated on a 6-point Likert scale (0 = always and 5 = never), and the PBQ includes four subscales: reflect impaired bonding (12 items), rejection and anger (7 items), anxiety about care (4 items), and risk of abuse (2 items) [40]. The maximum overall score is 125 points, and scores of 26–39 indicate “moderately impaired bonding”, whereas scores ≥ 40 indicate “severely impaired bonding” [41]. The PBQ is considered to have good reliability (Cronbach’s α = 0.92).

### Sample size

For a repeated-measures analysis of variance (ANOVA) with between-factors analysis, the following parameters were utilized: f = 0.25, alpha = 0.05, power = 0.80, number of groups = 2, number of measures = 5, and correlation among repeated measures = 0.5. Based on a previous mindfulness study, an attrition rate of 30% was expected [42], resulting in a target sample size of 102 participants.

### Randomization

Our study participants were randomly assigned in a 1:1 ratio to each group and divided into blocks 6, 8 and 12. A trained research assistant collected data using a computer-generated random number sequence previously prepared by the researcher, which was stored in an opaque sealed envelope. Each participant received an explanation of the study from a research assistant and was enrolled after providing written informed consent. They were asked to complete the study questionnaire, each taking approximately 20 min to complete. Participants received remuneration equivalent to 4–5 USD after each completed questionnaire to reduce attrition. Participants were blinded to their groups, and medical care providers, and the team of data monitoring and analysis were blinded to group assignments.

### Data analysis

Data obtained in the present study were analyzed using SPSS 26.0 (SPSS Inc. Chicago, IL, USA). Descriptive statistics (percentage, mean, standard deviation) were applied, and skewness and kurtosis values were calculated to assess whether numerical variables fit a normal distribution. Each dependent variable was z-standardized before the generalized estimating equation (GEE) and independent analyses to facilitate interpreting the effect. The dependent t-test was used to compare the mean PBQ scores between the two study groups, and GEE [43] was used to compare the mean EPDS, PRT and PSS scores between groups. Effect size (ES) was calculated using Cohen’s *d* to assess whether a change occurred between the pretest (T0) and posttests (T1, T2, T3, and T4) of group comparisons. A calculated ES of 0.2 was considered “small,” 0.5 was considered “moderate,” and ≥ 0.8 was considered “large” [44].

## Results

**Fig. 1:**
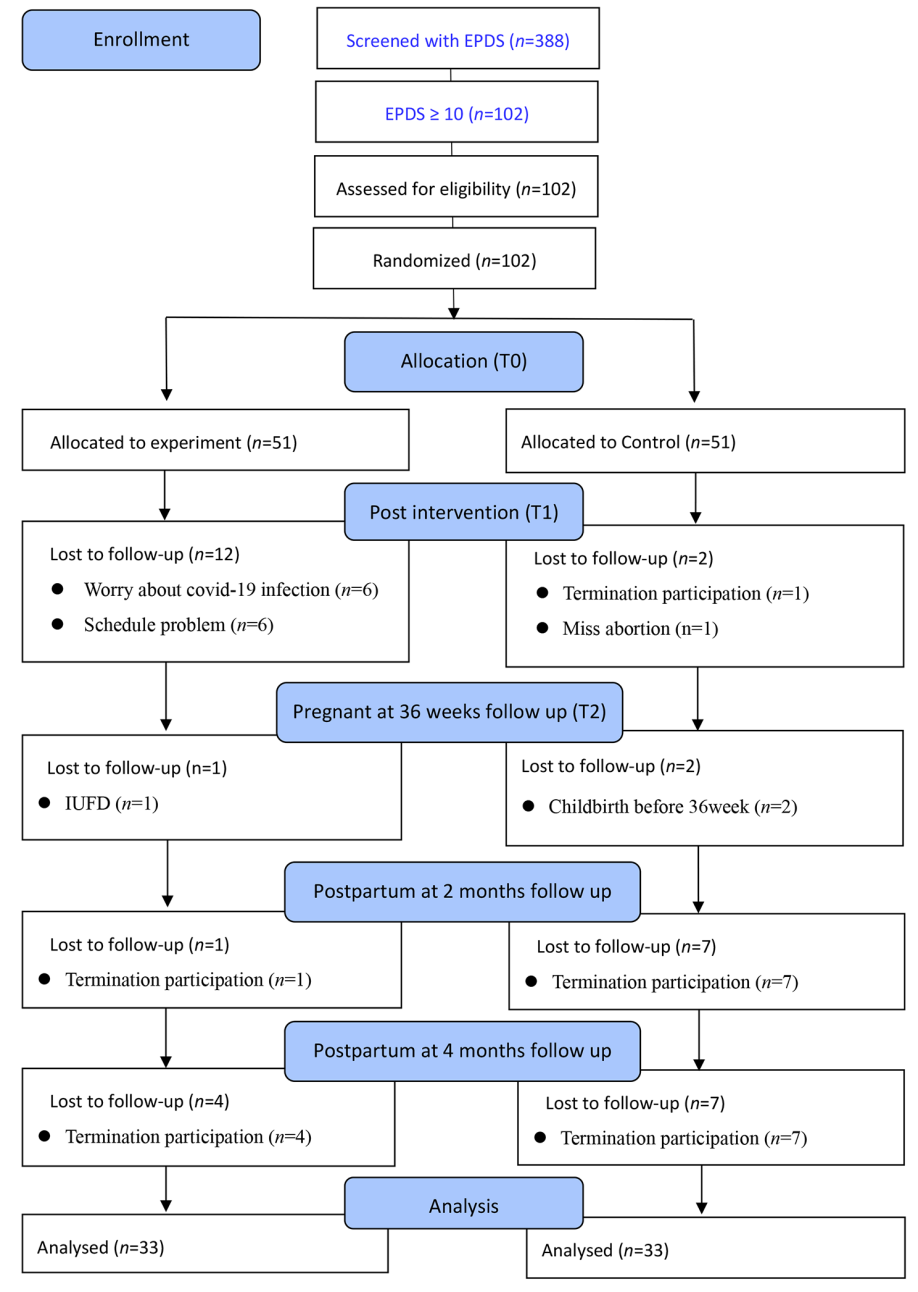
CONSORT diagram. Passage of participants through each trial stage

The baseline characteristics of participants were similar to those of those excluded. Consequently, we conducted a complete case analysis, which provides unbiased results compared to multiple imputation methods, particularly in cases where substantial amounts of missing data [45]. Figure 1 illustrates the recruitment process for participants in the CONSORT trial. A total of 102 participants were included, with 51 assigned in the intervention group and 51 to the control group. At T1 (post-intervention), 88 participants (86.27%) returned the questionnaire. At T2 (36 weeks of gestation), 85 participants (83.33%) returned the questionnaire. At T3 (2 months postpartum), 77 participants (75.49%) returned the questionnaire. At T4 (4 months postpartum), 66 participants (64.71%) returned the questionnaire. The final experimental and control groups each consisted of 33 participants each. In this study, several reasons were identified for participant withdrawal. These reasons included concerns about COVID-19 infection, scheduling problems, termination of participation, missed abortions, intrauterine fetal demise (IUFD), and childbirth before 36 weeks. The attrition rate for this study was 35.29%.

The participants were aged 23 to 45 years (33.20 ± 4.42), and most of them were educated at the university level or higher, married, employed, and primiparas. After the intervention, 76.56% of the mothers had a normal vaginal delivery; 51.61% of the infants born were female. Infants were mostly born at full-term, on average, with a mean birthweight of 2981.77 ± 289.67 g. After giving birth, 27.59% of the participants were taken care of by family members and 20.69% by a yuesao, while 51.72% stayed in a confinement care center. No significant differences in socioeconomic or obstetric characteristics were identified between the two groups (Table 2). Most participants in the experimental group reported continued practice of informal and/or formal mindfulness exercises after the 8-week program. The average attendance rate was 6.03 for eight sessions; among participants, 48 (72.73%) participated in > 6 sessions. The average engagement in formal mindfulness exercises was 5.67 times per week at T1, 4.2 times per week at T2, 2.56 times per week at T3, and 2.87 times per week at T4, with sessions lasting at least 30 min each.

**Table 2:** Comparison of participants the two study groups on in socioeconomic, obstetric characteristics and baseline questionnaire scores

Participant characteristic	Intervention group (*n* = 33)	Control group (*n* = 33)	*p*
Age: *M* (*SD*)	33.52 ± 4.91	32.88 ± 3.90	.56^c^
Level of education			
Junior college or below	4	2	.67^b^
University or above	29	31	
Marital Status			
Married	32	32	.75^b^
Not married	1	1	
Religiosity			
Religious	18	19	.80^a^
Not religious	15	14	
Employment status			
Employed	28	22	.09^a^
Unemployed	5	11	
Income			
Less than US$1500	17	12	.42^a^
US$1500–US$2999	11	13	
More than US$2999	5	8	
Pregnancy intention			.28^a^
Intentional	25	21	
Unintentional	8	12	
Parity			.57^a^
No prior births	27	22	
1 or more prior births	6	11	
Mode of Delivery°			.43^a^
Vaginal	25	22	
Cesarean	7	8	
Infant°			.81^a^
Male	15	15	
Female	17	15	
Infant baby weight°	3012.81 ± 296.11	2948.67 ± 283.85	.39^c^
Postpartum confinement care°			.25^a^
Family care	11	5	
Yuesao care	7	5	
Postpartum care centers	14	19	

^a^Chi-Square test; ^b^Fisher’s Exact Test; ^c^Independent t test; °Numbers may sum to less than 96 because of missing data; Significant at the *p* ≤ .05

### Depression in women with PMAD

**Fig. 2:**
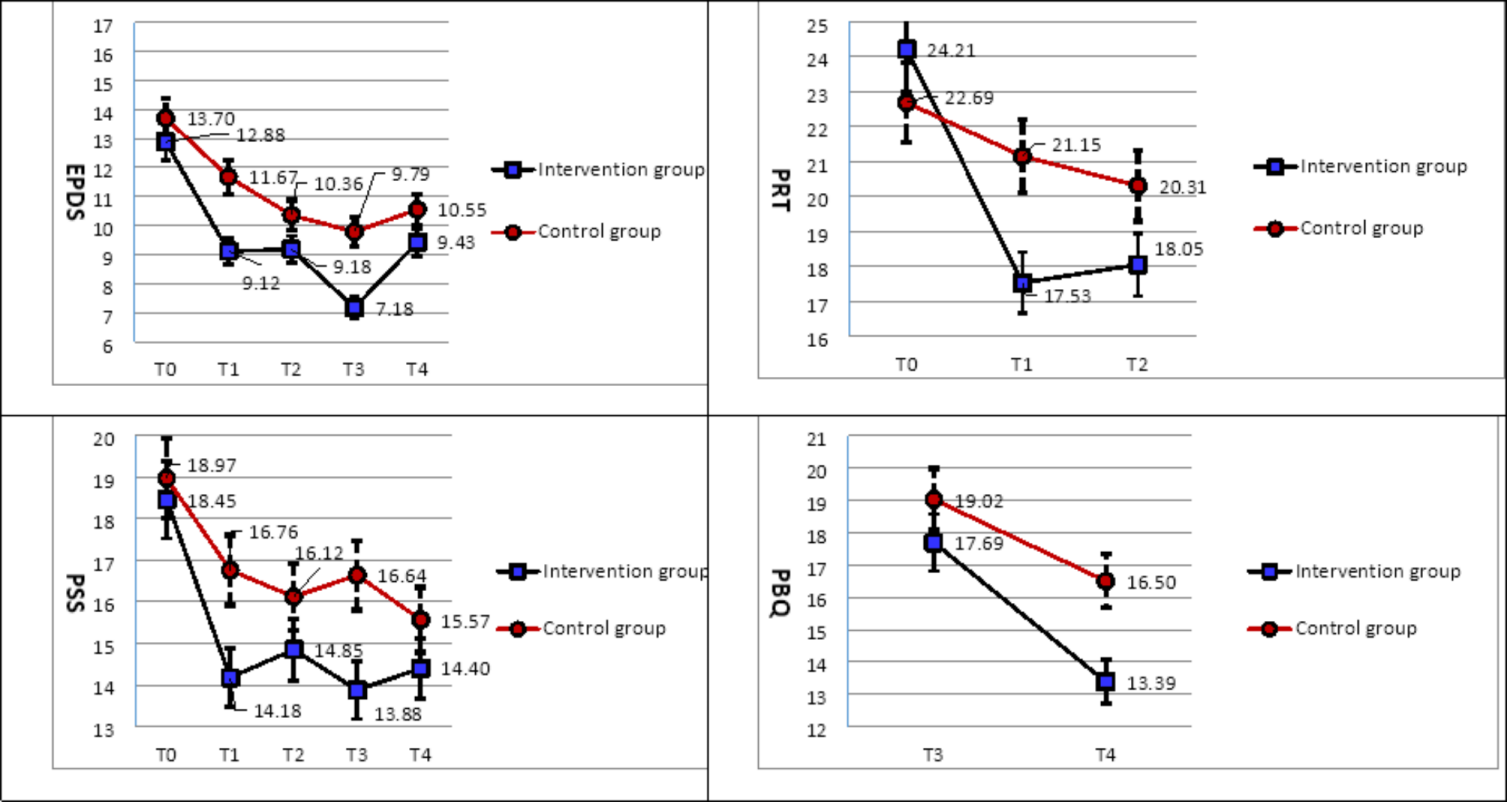
Changes in outcome measures over time T0 = Before the intervention; T1 = After the intervention; T2 = 36 weeks of gestation. T3 = 2 months postpartum; T3 = 4 months postpartum; EPDS = Edinburgh Postnatal Depression Scale; PSS = Perceived Stress Scale; PRT = Pregnancy-Related Thoughts Questionnaire; PBQ = Postpartum Bonding Questionnaire.

The baseline scores were 12.88 ± 2.99 in the intervention group and 13.70 ± 3.78 in the control group, indicating near “depression”. After participation in this program, the mean EPDS scores decreased with time, with a mean score of 9.12, 9.18, 7.18, and 9.43 in that order. This indicates “no depression” at all time points in the experimental group (Fig. 2). The experimental and control groups each consisted of 33 participants. This decrease was significant for the intervention group at T1 (*B* = − 0.69, *p* < .001, ES = 0.52) and T2 (*B* = 0.73, *p* < .001, ES = 0.22). After childbirth, EPDS z-scores decreased in the intervention group (*B* = 0.99, p < .001; *B* = − 0.69, p < .001), and the ES was medium to small at T3 and T4 (ES = 0.58; 0.29) (Table 3).

**Table 3:** Assessing the effects of the intervention on outcome measures using the GEE model or T-test (*n* = 66)

Variable	*B*	SE	95% CI		χ^2^	*p*
EPDS						
Group 1 vs. Group 0	−0.38	0.18	−0.72	0.01	3.72	0.052
T1 vs. T0	−0.69	0.97	−0.79	−0.41	38.07	< 0.001
T2 vs. T0	−0.73	0.11	−0.94	−0.51	43.53	< 0.001
T3 vs. T0	−0.99	0.11	−1.22	−0.77	76.22	< 0.001
T4 vs. T0	−0.69	0.14	−0.97	−0.42	24.81	< 0.001
PSS						
Group 1 vs. Group 0	−0.26	0.19	−0.10	0.62	1.95	0.163
T1 vs. T0	−0.62	0.12	−0.86	−0.38	25.76	< 0.001
T2 vs. T0	−0.62	0.12	−0.86	−0.38	26.32	< 0.001
T3 vs. T0	−0.66	0.15	−0.95	−0.38	20.63	< 0.001
T4 vs. T0	−0.76	0.18	−1.12	−0.41	17.74	< 0.001
PRT						
Group 1 vs. Group 0	−0.18	0.26	−0.69	0.33	0.48	0.487
T1 vs. T0	−0.84	0.17	−1.17	−0.51	25.13	< 0.001
T2 vs. T0	−0.85	0.18	−1.21	−0.49	21.19	< 0.001
PBQ						
Group 1 vs. Group 0 (T3)	–	–	–	–	–	0.869
Group 1 vs. Group 0 (T4)	–	–	–	–	–	0.554

The experimental and control groups each consisted of 33 participants; GEE = Generalized Estimating Equation; *B* = *B* values in GEE model; SE = Standard Error CI = Confidence Intervals; χ2 = Chi-square values; Group 1 = Experimental group; Group 0 = Control group; T0 = Before the intervention; T1 = After the intervention; T2 = 36 weeks of gestation. T3 = 2 month of postpartum; T4 = 4 month of postpartum; EPDS = Edinburgh Postnatal Depression Scale; PSS = Perceived Stress Scale; PRT = Pregnancy-Related Thoughts Questionnaire; PBQ = Postpartum Bonding Questionnaire; the EPDS, PSS, PRT, and PBQ variables were z-standardized before analysis

### Stress in women with PMAD

The baseline score for stress was 18.45 ± 4.91 for the intervention group and 18.97 ± 3.78 for the control group, indicating “moderate stress.” After participation in this program, the mean PSS-10 scores decreased with time; the mean score was 14.18, 14.85, 13.88, and 14.40 in that order, which indicated “mild stress” at all time points in the experimental group (Fig. 2). The GEE results showed reductions compared with the Z-standardized baseline. The decrease was significant for the intervention group at T1 (*B* = − 0.26, *p* < .001, ES = 0.53) and T2 (*B* = 0.62, *p* < .001, ES = 0.29). After childbirth, PSS z-scores decreased in the intervention group (*B* = 0.62, *p* < .001; *B* = − 0.66, *p* < .001,), and the effect size was medium to small at T3 and T4 (ES = 0.56; 0.21) (Table 3).

### Anxiety in women with PMAD

Over the course of the program was completed, the mean scores for the PRT level decreased. There was a mean score of 24.21 for the intervention group and 22.69 for the control group before this program began. In the intervention group, mean scores decreased to 17.53 and 18.05 points post-intervention (Fig. 2). In the GEE model, the mean PRT z-score decreased for the intervention group at T1 (*B* = 0.84, *p < .001*, ES = 0.74; large effect) and T2 (*B* = 0.85, *p* < .001; ES = 0.47; medium effect; Table 3).

### Mother–infant bonding in women with PMAD

After participating in the program, the average PBQ level score showed a downward trend. The average scores at T3 were 17.69 and 19.02 and at T4 were 13.39 and 16.50 for the intervention group and the control group, respectively (Fig. 2). Although the average scores of the experimental group at T3 and T4 were lower than those of the control group, these differences were nonsignificant. The effect size was small at all-time points (ES = 0.10; 0.25). Figure 2; Table 3 show the relevant data.

## Discussion

The objective of this study was to explore the effects of a mindfulness program on psychological distress and mother–infant bonding in perinatal women. The study findings support the feasibility, acceptability, and efficacy of the intervention among women with PMAD. Compared with the control group (standard antenatal care), the intervention group experienced significant reductions in self-reported depression, stress, and anxiety that persisted up to four months postpartum. Furthermore, after the intervention, the anxiety effect was large, except at 36 weeks of gestation (before delivery). Stress and depression effect sizes were moderate and significantly smaller after four months postpartum.

In our study, about 66% of the participants had moderate to high perceived stress. However, the mindfulness intervention resulted in not only a decrease in perceived stress but also an improvement in mental health indicators, such as symptoms of anxiety and depression. Individuals with increased mindfulness have been shown to have a more benign approach to cognitive assessments of stressful situations and use more adaptive coping strategies [46]. While participants in this study reported having less time available for formal mindfulness practice after childbirth due to caring for their newborns, they were still able to interact with other members of mindfulness teams through LINE. Supportive factors found in past research to sustain people’s mindfulness practice include “practical resources,” “time/routine,” “support from others,” and “attitudes and beliefs” [47]. Although we were unable to meet in person after our eight-week program ended, participants were still able to communicate and connect via LINE. In addition, the researchers provided information about perinatal mindfulness. Some participants also put their practice time and status into LINE, which may have helped some people to remember and maintain continuous practice during the postpartum period. Based upon our findings, we believe that it is important to constantly remind pregnant and postpartum women to maintain mindfulness practice after the completion of the 8-week course to support their mental health. For participants to sustain a formal mindfulness practice over an extended period of time, it was suggested that they should have the opportunity to meet or build mindfulness learning communities after the intervention [48].

It is important to note that our findings of improvement in psychological distress are consistent with those of several meta-analyses and review studies conducted in pregnancy and postpartum periods on women with or without pre-existing stress, depressive or anxiety disorders [49–51]. Nevertheless, one longitudinal study found that depression symptoms returned at 3, 9, and 12 months postpartum following the MBCP program [52]. In a recent study by Kral et al. [53], it was noted that mindfulness practice for at least 22 min each day for several months may lead to structural changes in the amygdala, which is an important part of the brain involved in fear and anxiety. Accordingly, the discrepancy between our findings and those of Lönnberg et al. [52] may also be due to their inability to engage participants in mindfulness practice after delivery, resulting in insignificant effects after 3 months postpartum. Two meta-analyses of randomized clinical trials comparing mindfulness interventions for perinatal women found small to medium effect sizes for anxiety and depression and a small effect size for stress in pre- and post-analyses. However, only a few follow-up studies remained significant over time [54, 55]. Our study’s results were similar to previous studies until two months postpartum. However, stress and depression were smaller at 36 weeks of pregnancy and four months postpartum. Previous studies have found that peripartum women’s stress and depression symptoms decrease in the second trimester and then increase again in late pregnancy [56]. The intervention time for this study was 19.91 ± 5.61 weeks of gestation. Therefore, future studies should set the intervention time in the third trimester. Additionally, special attention should be given to monitoring the emotional well-being of women before childbirth.

Bögels et al. [56] suggested that mindfulness skills can reduce maternal stress, thereby reducing mothers’ fight or flight responses to parenting stress and improving their ability to make situationally aware decisions; thus, past studies have found that prenatal mindfulness improved the mother–infant relationship [57]. However, although the experimental group’s score for PBQ was lower than the control group’s score in our study, this difference was not significant. This may be due to our participants not having postpartum bonding disorder before the intervention. In our study, 72.41% of the participants were in a postpartum care center or under yuesao care during the perinatal period when mothers are often separated from their babies to enable the women to get more rest [58]. This may lead to an initial high score for postpartum bonding disorder and then a low score. However, the experimental group’s score showed a higher drop. Future psychological interventions could take advantage of this evidence on the mother–infant bond and cultural differences.

There are several limitations to this study. In spite of the fact that the initial sample size was sufficient for the purposes of this study, there was a high attrition rate (35.29%). The effect size may be overestimated, but there was no significant difference in attrition between the intervention and control groups. To capture the ongoing effects of the course more accurately, future studies should focus on strategies to improve questionnaire retention. The study also had the limitation that participants in this study were women with a predisposition for depression, using an EPDS screening score of 10 or higher; therefore, these results cannot be generalized to all women with PMAD. Thirdly, self-reported outcome measures were utilized to assess depression, stress, anxiety, quality of mother-infant bonding, and mindfulness practice time. The fourth limitation of this study is that the participants were Taiwanese and the study was conducted during the COVID-19 pandemic, which might limit their generalizability to other cultures and periods. Future studies should seek to replicate these results with a more diverse sample and a typical time context.

## Conclusion

Previously published research findings on prenatal mindfulness in Western countries were extended to Asian mothers in Taiwan, demonstrating that long-term mental health improved for women. The results of our study demonstrate that women who completed a high-dose mindfulness intervention for eight weeks had lower levels of depression, maintaining non-clinical levels of depressive symptomatology. As a result of these findings, empirical evidence supports that mindfulness practices alleviate PMAD among perinatal women. It is thus incumbent upon medical professionals to provide support to these women to mitigate the impacts of stressors on their parenting life transition. Such stressors may be addressed by mindfulness programs for perinatal women. Future research might consider including an additional mediation analysis with perceived stress. As part of the process of improving the maintenance of regular practice of mindful exercises at the end of the intervention, it is necessary to examine the mediation approaches and test these effects. Furthermore, the intervention should be offered at multiple centers with larger sample sizes to ensure that these findings are applicable.

## Data Availability

Data used and analyzed in the current study are available from the corresponding author upon reasonable request.

## Abbreviations

PMAD: Perinatal mood and anxiety disorder
MBCP: Mindfulness based childbirth and parenting
EPDS: Edinburgh postnatal depression scale
PSS: Perceived stress scale
PRT: Pregnancy-related thoughts questionnaire
PBQ: Postpartum bonding questionnaire
